# Inhibition of ZEB1 expression induces redifferentiation of adult human β cells expanded *in vitro*

**DOI:** 10.1038/srep13024

**Published:** 2015-08-12

**Authors:** Elad Sintov, Gili Nathan, Sarah Knoller, Metsada Pasmanik-Chor, Holger A. Russ, Shimon Efrat

**Affiliations:** 1 Department of Human Molecular Genetics and Biochemistry, Sackler School of Medicine, Tel Aviv University, Tel Aviv, Israel.; 2 Bioinformatics Unit, George Wise Faculty of Life Sciences, Tel Aviv University, Tel Aviv, Israel

## Abstract

*In-vitro* expansion of functional adult human β-cells is an attractive approach for generating insulin-producing cells for transplantation. However, human islet cell expansion in culture results in loss of β-cell phenotype and epithelial-mesenchymal transition (EMT). This process activates expression of ZEB1 and ZEB2, two members of the zinc-finger homeobox family of E-cadherin repressors, which play key roles in EMT. Downregulation of *ZEB1* using shRNA in expanded β-cell-derived (BCD) cells induced mesenchymal-epithelial transition (MET), β-cell gene expression, and proliferation attenuation. In addition, inhibition of ZEB1 expression potentiated redifferentiation induced by a combination of soluble factors, as judged by an improved response to glucose stimulation and a 3-fold increase in the fraction of C-peptide-positive cells to 60% of BCD cells. Furthermore, *ZEB1* shRNA led to increased insulin secretion in cells transplanted *in vivo.* Our findings suggest that the effects of ZEB1 inhibition are mediated by attenuation of the miR-200c target genes *SOX6* and *SOX2*. These findings, which were reproducible in cells derived from multiple human donors, emphasize the key role of ZEB1 in EMT in cultured BCD cells and support the value of ZEB1 inhibition for BCD cell redifferentiation and generation of functional human β-like cells for cell therapy of diabetes.

Beta-cell replacement by transplantation represents an attractive approach for treatment of diabetes, however it is limited by shortage of human tissue donors. Potential new sources of β-like cells include pluripotent stem cells induced to differentiate into insulin-producing cells^1^,^2^,^3^, nuclear reprogramming of non-β differentiated cell types^4^, and *in-vitro* expansion of adult human islet cells from cadaver donors^5^. Proliferation of β-cell-derived (BCD) cells cultured from multiple human donors has been shown by cell lineage-tracing to be associated with loss of β-cell phenotype^6^ and epithelial-mesenchymal transition (EMT)^7^. EMT occurs during embryogenesis, as well as cancer progression, and involves loss of epithelial cell polarity, severance of intercellular adhesive junctions, and acquisition of a motile mesenchymal phenotype^8^,^9^. A number of signaling pathways, including WNT-β-catenin, TGFβ-SMAD2/3, Hedgehog-GLI, and Jagged1-NOTCH^10^,^11^,^12^,^13^, have been implicated in upregulating the expression of transcription factors important for EMT, such as SNAI1, SNAI2 (SLUG), TWIST, ZEB1 (deltaEF1), and ZEB2 (SIP1)^14^,^15^,^16^, all of which downregulate E-cadherin expression by repression of *CDH1*^13^. BCD cells, which constitute ~40% of human islet cell cultures^6^, maintain open chromatin structure at β-cell genes^17^ and can be redifferentiated in response to a combination of soluble factors termed Redifferentiation Cocktail (RC)^18^. However, RC treatment leads to redifferentiation of only part of BCD cells. We therefore investigated approaches for improving redifferentiation. In recent reports, the NOTCH^19^ and WNT^20^ pathways have been shown to be activated during dedifferentiation of BCD cells. Inhibition of the NOTCH pathway mediator HES1^21^, and WNT pathway mediator β-catenin^20^, resulted in enhanced BCD cell redifferentiation. Here we evaluated the effect of ZEB1 inhibition on promoting BCD cell redifferentiation following expansion in culture. ZEB1 and ZEB2 are members of the zinc-finger homeobox (ZFH) family of repressors, which activate EMT by binding to E-box elements in the *CDH1* promoter and suppressing its activity^22^. ZEB1 also promotes EMT by repressing expression of basement membrane components and cell polarity proteins. In addition, ZEB1 has been found to trigger a micro RNA (miR)-mediated double-negative feedback loop that stabilizes EMT. ZEB1 directly suppresses expression of the miR-200 family, and is also one of the predominant targets of these miRs^23^,^24^,^25^,^26^,^27^. Here we show that ZEB1 expression is activated in expanded human islet cells. Inhibiting its expression by shRNA leads to BCD cell growth arrest, mesenchymal-epithelial transition (MET), and redifferentiation. ZEB1 inhibition synergizes with RC treatment, resulting in enhanced BCD cell redifferentiation. Our findings suggest that the ZEB1/miR-200 feedback loop may mediate the effects of ZEB1 inhibition.

## Results

### Induction of ZEB expression during islet cell dedifferentiation

**Figure 1:**
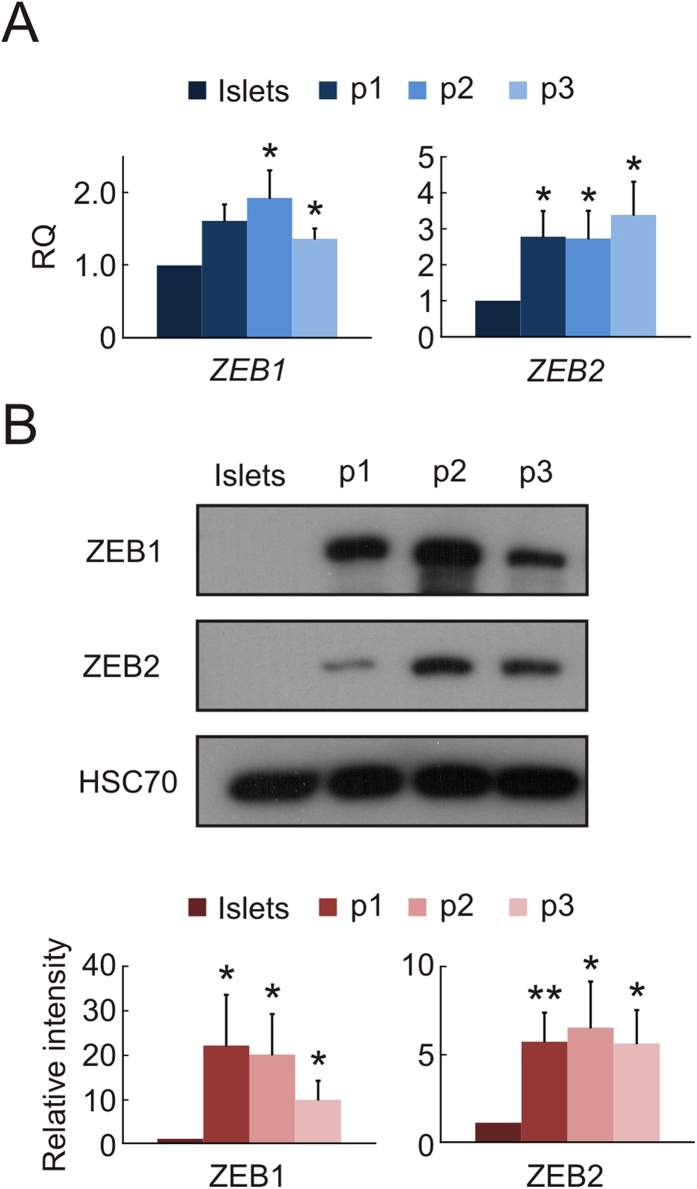
Induction of ZEB expression during islet cell dedifferentiation. (**A**) qPCR analysis of RNA extracted from expanded islet cells at the indicated passages. Values are mean ± SE, relative to uncultured islets (n = 3–6 donors), and normalized to *GAPDH*, or *RPLPO* and *TBP*. *p < 0.05. (**B**) Immunoblotting analysis of protein extracted from expanded islet cells at the indicated passages (cropped blot). HSC70, heat-shock cognate 70. Values are mean ± SE, relative to uncultured islets (n = 3–7 donors). *p < 0.05, **p < 0.01.

*ZEB1* and *ZEB2* transcripts were significantly upregulated in islet cells during the first 3 weeks of culture, as revealed by qPCR analyses (Fig. 1A). Immunoblotting revealed that both ZEB1 and ZEB2 were upregulated during the first week of culture, and their high levels were maintained thereafter during cell propagation (Fig. 1B).

### ZEB1 inhibition induces insulin expression and reduces BCD cell proliferation

**Figure 2:**
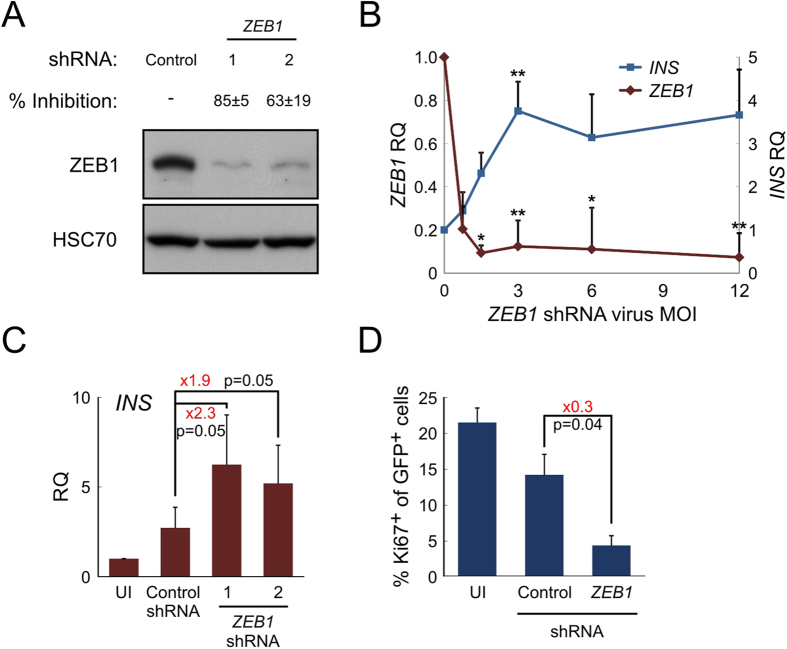
ZEB1 inhibition restores insulin expression in expanded islet cells and blocks BCD cell replication. (**A**) *ZEB1* inhibition by shRNA. Immunoblotting analysis of expanded islet cells infected at passage 6 with lentiviruses expressing *ZEB1* (shRNA#1, TRCN-17563; shRNA#2, TRCN-17566) or control shRNA and analyzed 7 days later (cropped blot). Percent inhibition is mean ± SE (n = 3 donors; p = 1 × 10^−5^ for shRNA#1, p = 0.004 for shRNA#2). (**B**) qPCR analysis of expanded islet cells infected at passages 4–6 with increasing amounts of *ZEB1* shRNA#1 or control shRNA lentiviruses. Values are mean ± SE (n = 3–5 donors), normalized to human *RPLPO* and *TBP*, relative to cells infected with control shRNA at MOI of 3 (RQ = 1). *p ≤ 0.05, **p ≤ 0.01, relative to cells infected with control shRNA. (**C**) qPCR analysis of expanded islet cells infected at passages 4–6 with *ZEB1* or control shRNA lentiviruses at MOI 3:1. UI, uninfected cells. Values are mean ± SE (n = 3–5 donors) and normalized to human *RPLPO* and *TBP*. (**D**) Immunofluorescence analysis of expanded islet cells, labeled with eGFP and infected at passages 6–7 with lentiviruses expressing *ZEB1* or control shRNAs, using antibodies for Ki67 and eGFP. Values are mean ± SE (n = 3 donors), based on counting >200 cells for each donor.

To evaluate the possibility for reversing the effects induced by ZEB proteins in cells which underwent EMT, shRNA was employed to block *ZEB* expression. Infection of expanded islet cells with two different *ZEB1* shRNA lentiviruses reduced ZEB1 protein levels by up to 85 ± 5% (Fig. 2A), while significantly elevating insulin transcript levels, relative to cells infected with a control shRNA (Fig. 2B,C). Among the two *ZEB1* shRNAs, shRNA#1 was chosen for further experiments due to its higher efficiency. The levels of insulin transcripts were inversely proportional to the levels of *ZEB1* transcripts, which were a function of the MOI of the *ZEB1* shRNA virus (Fig. 2B). *ZEB2* shRNA reduced ZEB2 protein levels by up to 65 ± 40% (see Supplementary Fig. S1A online). However, subsequent analyses revealed that ZEB2 inhibition did not significantly affect *INS* transcript levels (see Supplementary Fig. S1B online). Therefore, further detailed analyses focused primarily on ZEB1 manipulation.

To determine the effect of ZEB1 inhibition on BCD cell replication, cells dissociated from isolated human islets were infected with two lentiviruses, one expressing Cre recombinase under control of the insulin promoter and the other, a reporter cassette with the structure cytomegalovirus (CMV) promoter-loxP-Stop-loxP-eGFP. In this system, eGFP expression is blocked by a loxP-flanked DNA fragment. Removal of the block specifically in β-cells activates eGFP expression during the initial days of culture, when the insulin promoter is still expressed, resulting in labeling of about 50% of β-cells. Labeled cells were then expanded, transduced with shRNA vectors, and stained for Ki67. The fraction of Ki67^+^ cells among eGFP-labelled BCD cells was significantly reduced about 3-fold following *ZEB1* shRNA treatment, compared to control shRNA (Fig. 2D). To evaluate cell mortality rates 8–10 days following shRNA lentivirus infection, cells were analyzed by TUNEL assay. The *ZEB1* shRNA treatment did not result in a significant increase in apoptosis, compared with control shRNA (0.43 ± 0.12% and 0.47 ± 0.25% TUNEL-positive cells for control and *ZEB1* shRNAs, respectively, compared with 0.13 ± 0.15% for uninfected cells).

### Redifferentiation of BCD cells induced by ZEB1 inhibition

**Figure 3:**
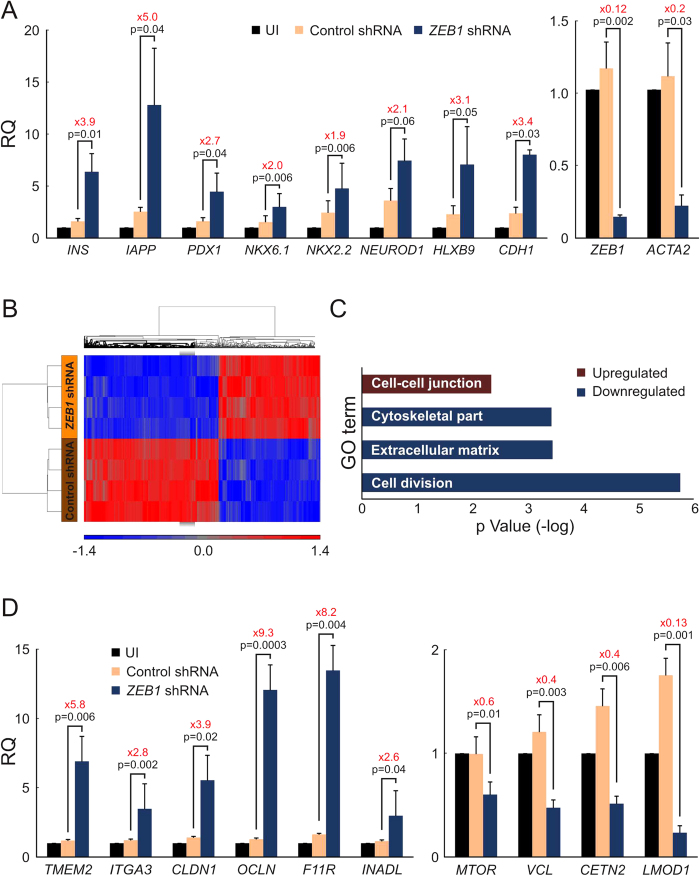
Redifferentiation of BCD cells induced by ZEB1 inhibition. Expanded islet cells transduced with the lineage tracing lentiviruses were sorted by FACS at passages 2–3. eGFP-labeled BCD cells were then grown until passages 6–7 and transduced with lentiviruses expressing *ZEB1* or control shRNAs. RNA was extracted 7 days later. (**A**) qPCR analysis. (**B,C**) cDNA microarray analysis (n = 4 donors). (**B**) Hierarchical cluster analysis. Scale represents fold change relative to average values of samples from the other group. (**C**) DAVID functional annotation. (**D**) qPCR validation of genes listed in Table 4. (**A,D**) Values are mean ± SE (n = 3–4 donors) relative to UI and normalized to human *RPLPO* and *TBP*. UI, uninfected cells.

Previous findings demonstrated that restoration of β-cell gene expression in expanded islet cells represents BCD cell redifferentiation, rather than *de-novo* differentiation of non-BCD cells^18^,^20^,^21^. To determine whether the observed changes induced in expanded islet cells by ZEB1 inhibition were due primarily to BCD cell redifferentiation, islet cells were labeled with the lineage tracing vectors, and BCD cells were sorted at passages 2–3 to 85–90% purity. ZEB1 inhibition in these cells led to a significant increase in transcripts encoding insulin, IAPP, and β-cell transcription factors, relative to the control shRNA, which was comparable to the increase observed in mixed islet cell cultures (Fig. 3A, left). *CDH1* transcripts encoding the epithelial marker E-cadherin were upregulated 3.4-fold, while those for the mesenchymal marker ACTA2 (αSMA) were downregulated 5-fold, compared with the control shRNA, indicating the occurrence of MET (Fig. 3A, right). In contrast to INS transcripts, no significant increase was observed in transcripts encoding other islet hormones (see Supplementary Fig. S2 online).

To assess global changes in gene expression following ZEB1 inhibition, cDNA microarray analyses were performed on RNA extracted from sorted BCD cells treated with *ZEB1* or control shRNAs. A total of 380 genes were upregulated, while 287 genes were downregulated (fold change >1.5; pFDR < 0.05) (Fig. 3B). Supplementary Table S4 lists the genes with the largest changes in expression. DAVID functional annotation revealed significant changes in 1291 downregulated genes (fold change >1.25; pFDR < 0.05) sharing the annotation terms cell division, extracellular matrix, and cytoskeletal part, consistent with the effects of ZEB1 on cell proliferation and EMT (Fig. 3C). 1327 upregulated genes (fold change >1.25; pFDR < 0.05) shared the cell-cell junction ontology term, implying a restoration of epithelial phenotype^28^ (see Supplementary Table S5 for a full list of all significant GO terms). To validate the microarray results, ten genes (Table 1) were analyzed by qPCR (Fig. 3D). Among the genes upregulated upon ZEB1 inhibition, *OCLN, CLDN1, F11R* and *INADL* encode epithelial tight junction proteins. *F11R* and *INADL* are direct ZEB1 targets, containing a ZEB1 binding site in their promoter region^28^. Among downregulated genes, *MTOR* encodes a protein contributing to cell proliferation, while *VCL* encodes a protein involved in cell-cell and cell-matrix junctions.

### Human C-peptide release from cells treated with ZEB1 shRNA

**Figure 4:**
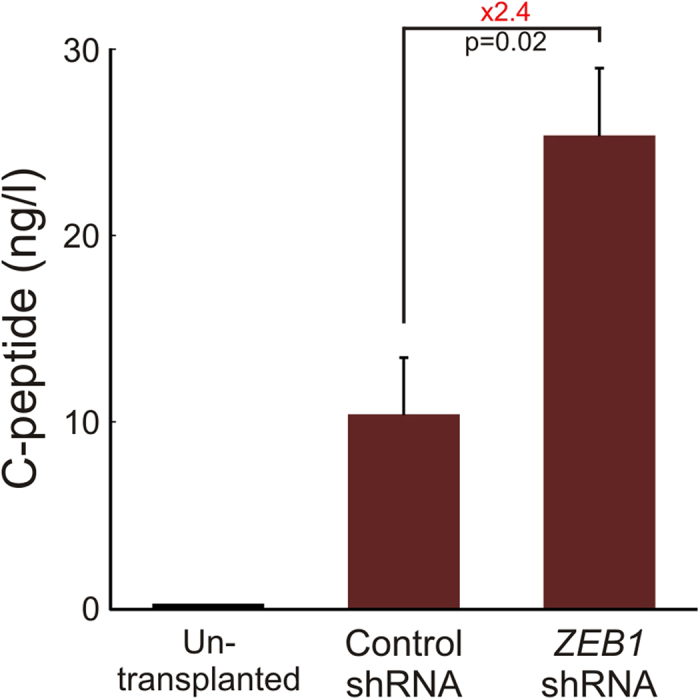
ZEB1 inhibition induces human C-peptide release from expanded islet cells transplanted into NSG mice. 1–2 × 10^6^ expanded islet cells infected at passages 5–6 with lentiviruses expressing *ZEB1* or control shRNAs were transplanted 7 days later under the renal capsule of >12-w male NSG mice. Serum samples were collected from fed mice 42–57 days later and assayed for human C-peptide by ELISA. Values are mean ± SE (n = 4 mice transplanted with *ZEB1* shRNA-treated cells, and 3 mice transplanted with control shRNA-treated cells, from 3 different donors).

Expanded islet cells infected with *ZEB1* or control shRNA were transplanted under the kidney capsule of NSG mice. A 2.4-fold increase in serum human C-peptide levels was observed in fed mice transplanted with *ZEB1* shRNA-treated cells, compared to mice transplanted with control shRNA-treated cells (Fig. 4).

### Synergistic redifferentiation effect of ZEB1 inhibition and soluble factors

**Figure 5:**
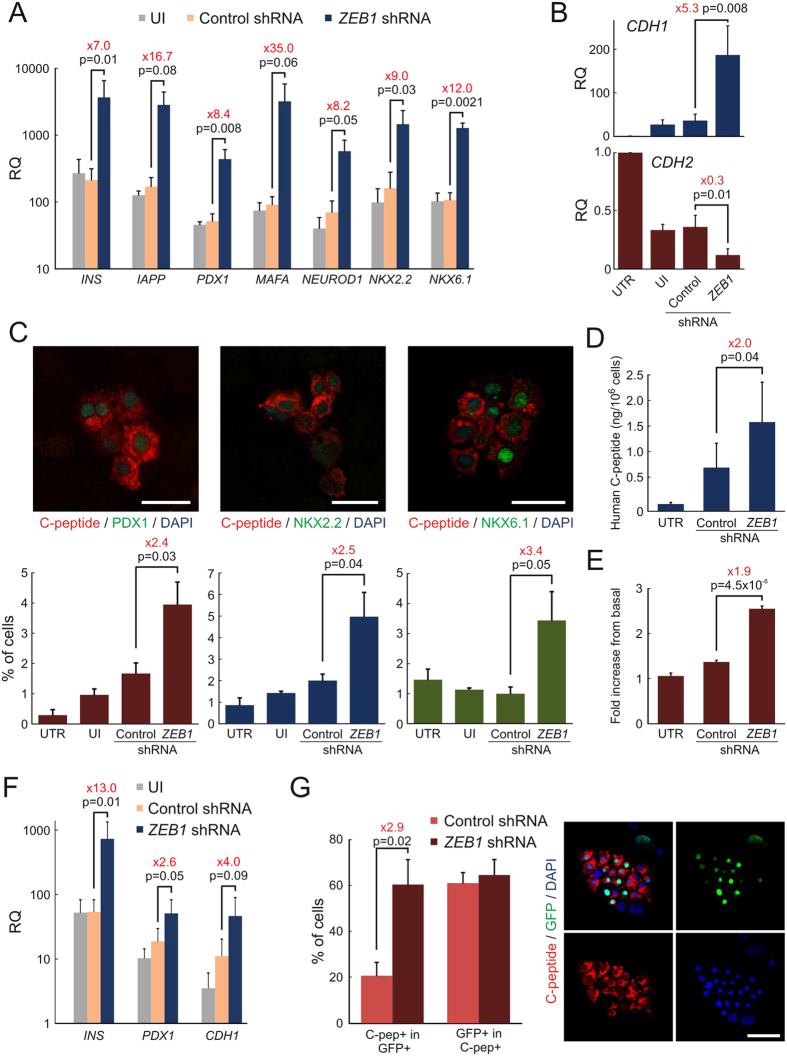
Synergistic effect of ZEB1 inhibition and RC treatment. (**A,B**) qPCR analysis of transcripts encoding β-cell proteins (**A**) and cell adhesion molecules (**B**) in RNA extracted from expanded islet cells 7 days following infection at passages 5–7 with lentiviruses expressing *ZEB1* or control shRNAs, and an 8-day treatment with RC. Values are mean ± SE (n = 3–5 donors) relative to untreated cells (UTR; RQ = 1) and normalized to human *RPLPO* and *TBP*. UI, uninfected cells treated with RC. (**C**) Immunofluorescense analysis of expanded islet cells, 7 days following infection at passages 5–7 with lentiviruses expressing *ZEB1* or control shRNAs, and a 4-day treatment with RC. Percent of double-positive cells are mean ± SE (n = 3 donors), based on counting >500 cells from each donor. Cells treated with *ZEB1* shRNA are displayed. Bar = 25 μm. (**D**) Human C-peptide content of expanded islet cells 7 days following infection at passages 6–7 with lentiviruses expressing *ZEB1* or control shRNAs, and a 4-day treatment with RC. Values are mean ± SE (n = 3 donors). (**E**) Glucose-induced insulin secretion from expanded islet cells 7 days following infection at passages 6–7 with lentiviruses expressing *ZEB1* or control shRNAs, following a 4-day RC treatment. Values are mean ± SE (n = 3–4 donors) of human C-peptide released in the presence of 16.7 mM glucose, relative to 0 mM glucose. (**F**) qPCR analysis of BCD cells sorted at passages 2–3, infected at passages 6–7 with lentiviruses expressing *ZEB1* or control shRNAs, and treated 7 days later with RC for 4 days. Values are mean ± SE (n = 3–4 donors) relative to UTR (RQ = 1) and normalized to human *RPLPO* and *TBP*. (**G**) Immuonflourescence analysis of eGFP-labeled islet cells infected at passage 5–7 with *ZEB1* or control shRNA lentiviruses and treated 7 days later with RC for 4 days. Values are mean ± SE (n = 3 donors), based on counting >300 cells in all donors. Bar = 50 μm.

Previous work has demonstrated the ability of BCD cells to undergo significant redifferentiation in response to a combination of soluble factors termed Redifferentiation Cocktail (RC) in serum-free medium^18^. ZEB1 silencing potentiated the effect of 8-day RC treatment on transcripts encoding the β-cell proteins insulin and IAPP, as well as the β-cell transcription factors PDX1, NEOROD1, NKX2.2, NKX6.1 and MAFA, 7-35-fold (Fig. 5A). ZEB1 silencing also potentiated the RC-induced downregulation of *CDH2* transcripts, encoding N-cadherin, while *CDH1* transcripts were significantly upregulated (Fig. 5B). In contrast to ZEB1 inhibition, *ZEB2* shRNAs had only a marginal effect on *INS* transcript induction by RC (see Supplementary Fig. S1B online), indicating that ZEB1 was the main effector in this system. Immunostaining following a 4-day RC treatment revealed that *ZEB1* shRNA induced a 2.4-fold increase in the fraction of C-peptide/PDX1 double-positive cells, compared to cells treated with RC and control shRNA (Fig. 5C and Supplementary Fig. S3 online). The fraction of C-peptide^+^ cells co-stained for the β-cell transcription factors NKX2.2 or NKX6.1 also increased significantly in *ZEB1* shRNA-treated cells. Analysis of extracts of shRNA-infected cells following RC treatment revealed a 2-fold increase in C-peptide content in *ZEB1* shRNA-treated cells, compared to cells treated with control shRNA (Fig. 5D). Considering the fraction of C-peptide^+^ cells in the cell population (Fig. 5C), the content achieved with the combined treatment represents an estimated 1% of C-peptide content of normal islet β cells. Glucose induced a 2.6-fold increase in insulin release in cells redifferentiated with the combined treatment, compared with a 1.3-fold increase in cells treated with RC and control shRNA (Fig. 5E). The higher secretory response to glucose induced by the combination of ZEB1 inhibition and RC treatment was due to both a lower basal secretion (100 ± 11 pg/10^6^ cells per hour, compared to 148 ± 73 pg/10^6^ cells treated with RC and control shRNA), as well as an elevated glucose-stimulated secretion (286 ± 65 pg/10^6^ cells per hour, compared to 240 ± 134 pg/10^6^ cells treated with RC and control shRNA), which are both manifestations of increased β-cell differentiation. The synergistic effect of RC and *ZEB1* shRNA observed in cultures of mixed islet cells was reproduced in FACS-sorted BCD cells, as manifested by a 13-fold increase in insulin transcript levels, compared to cells treated with RC and control shRNA (Fig. 5F). *PDX1* and *CDH1* transcripts were also upregulated by ZEB1 inhibition, compared to control shRNA. Co-staining of eGFP and C-peptide following ZEB1 inhibition and RC treatment revealed that the combined treatment led to redifferentiation of 60% of BCD cells (Fig. 5G). Given the cell labeling efficiency with eGFP (~50%), the finding that >50% of C-peptide^+^ cells were eGFP^+^ suggests that BCD cells are the predominant source of insulin-expressing cells induced by the combined treatment, confirming our previous findings with RC treatment alone^18^. The 60% BCD cell redifferentiation efficiency is the highest achieved so far, compared with previously reported treatments^18^,^20^,^21^,^29^.

### ZEB1 inhibition in BCD cells is associated with miR-200c activation

**Figure 6:**
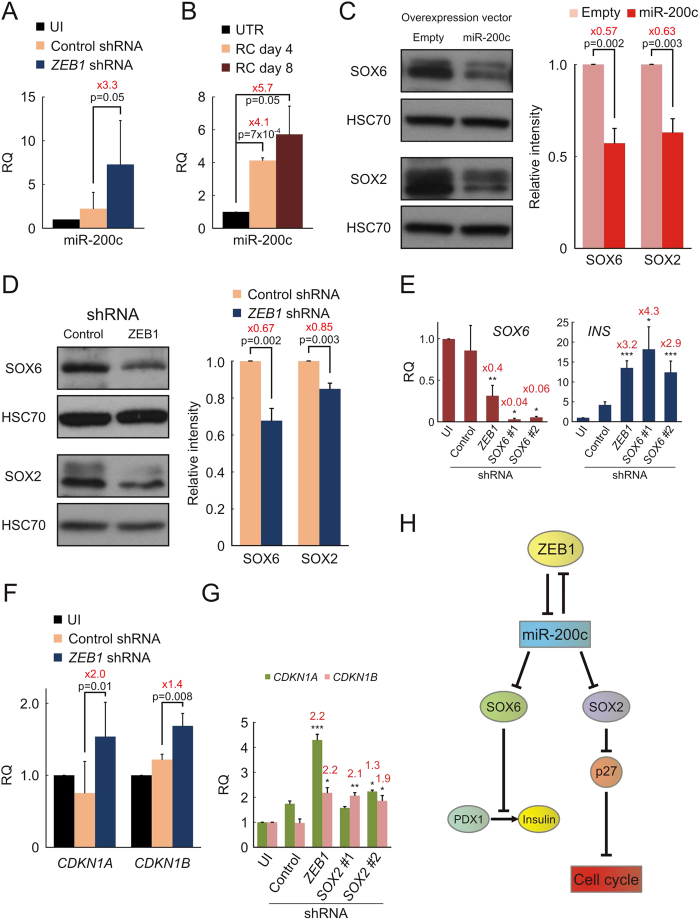
Insulin expression and cell proliferation in expanded islet cells is regulated by the ZEB1/miR-200 negative feedback loop. (**A**) qPCR analysis of miR-200c expression in FACS-sorted BCD cells infected at passages 6–7 with lentiviruses expressing *ZEB1* or control shRNAs. UI, uninfected cells. Values are mean ± SE (n = 3 donors), relative to UI, and normalized to human U6 snRNA and miR-24. (**B**) qPCR analysis of changes in miR-200c levels in expanded islet cells treated at passages 5–7 with RC for the indicated times. Values are mean ± SE (n = 3 donors), relative to UTR, and normalized to human U6 snRNA and miR-24. (**C,D**) Immunoblotting analysis of protein extracted from expanded islet cells infected at passages 3–4 with retroviruses expressing human miR-200c or an empty vector (**C**), or at passages 5–6 with lentiviruses expressing *ZEB1* or control shRNAs (**D**) (cropped blots). HSC70, heat-shock cognate 70. Values are mean ± SE (n = 4 donors). (**E**) Effect of *SOX6* shRNA on insulin expression. qPCR analysis of expanded islet cells infected at passages 5–6 with lentiviruses expressing *SOX6* shRNA#1 (TRCN-017988), shRNA#2 (TRCN-017991), or control shRNA. Values are mean ± SE (n = 3–4 donors), relative to UI, and normalized to human *RPLPO.* Indicated fold change and p-values are relative to control shRNA. *p < 0.05, **p < 0.01, ***p < 0.005. (**F**) qPCR analysis of FACS-sorted BCD cells infected at passages 6–7 with lentiviruses expressing *ZEB1* or control shRNAs. Values are mean ± SE (n = 3 donors), relative to UI, and normalized to human *RPLPO* and *TBP*. (**G**) Effect of *SOX2* shRNAs on expression of cell cycle regulators. qPCR analysis of expanded islet cells infected at passage 6 with lentiviruses expressing *SOX2* shRNA#1 (TRCN-010772), shRNA#2 (TRCN-355694), or control shRNA. Values are mean ± SE (n = 3–4 donors), relative to UI, and normalized to human *RPLPO.* Indicated fold change and p-values are relative to control shRNA. *p < 0.05, **p < 0.01. *SOX2* shRNAs inhibited *SOX2* transcript levels by up to 28 ± 22% (n = 4 donors, p = 0.06). (**H**) A proposed model for mediation of ZEB1 effects on PDX1-induced insulin gene expression and cell cycle through miR-200c, SOX6, and SOX2.

The occurrence of a double-negative feedback loop between the ZEB family of transcription factors and the miR-200 family of miRs in EMT and tumor invasion has been reported in recent years^23^,^24^,^25^,^26^,^27^. Using a miRNA microarray we have recently identified the miR-200 family among miRNAs highly downregulated during BCD cell dedifferentiation^29^. We hypothesized that the ZEB1/miR-200 feedback loop may account in part for the ZEB1 effects on EMT, cell proliferation, and inhibition of insulin expression in expanded BCD cells. To investigate a possible involvement of miR-200, RNA from sorted eGFP-labeled BCD cells treated with *ZEB1* shRNA was analyzed by qPCR for miR-200 expression. miR-200c was found to be significantly upregulated 3.3-fold following ZEB1 inhibition (Fig. 6A), while miR-200a and miR-200b were not significantly affected (see Supplementary Fig. S4 online). Additionally, treatment of expanded islet cells with RC stimulated a similar increase in miR-200c levels (Fig. 6B), suggesting that miR-200c played a role in BCD cell redifferentiation induced by RC. We therefore studied the effect of miR-200c overexpression on redifferentiation of expanded islet cells using a retrovirus vector expressing pre-miR-200c. miR-200c levels in transduced cells were upregulated by 770 ± 550 fold (mean ± SE; n = 3; p = 0.03). Expression of SOX6 and SOX2, two transcription factors encoded by transcripts targeted by miR-200c^30^,^31^, was downregulated in expanded islet cells overexpressing miR-200c by 43% and 37%, respectively (Fig. 6C). ZEB1 inhibition in expanded islet cells had a similar effect on these two miR-200c targets: SOX6 expression was significantly decreased by 33%, while SOX2 was downregulated by 15%, (Fig. 6D). SOX6 was shown to inhibit transactivation of the insulin gene by PDX1^32^, suggesting that its downregulation by miR-200c may stimulate insulin gene expression. Indeed, downregulation of *SOX6* transcripts in expanded islet cells using *SOX6* shRNA resulted in an increase in *INS* transcript levels similar to that induced by *ZEB1* shRNA (Fig. 6E). SOX2 plays a role in stimulating cell proliferation and dedifferentiation, and has been shown to facilitate G1/S transition by suppression of p21^Cip1^ and p27^Kip1^, two key cyclin/CDK inhibitors^33^. In support of this mechanism, p21^Cip1^ (*CDKN1A*) and p27^Kip1^ (*CDKN1*B) transcripts were significantly elevated in *ZEB1* shRNA-treated BCD cells, compared to cells treated with control shRNA (Fig. 6F). Furthermore, downregulation of SOX2 expression in expanded islet cells using *SOX2* shRNA resulted in an increase in *CDKN1*B transcript levels similar to that induced by *ZEB1* shRNA (Fig. 6G). Taken together, these findings suggest possible mechanistic links between ZEB1 downregulation, insulin gene expression, and growth arrest during BCD cell redifferentiation (Fig. 6H).

## Discussion

Our results demonstrate that ZEB proteins are induced during human islet cell expansion and dedifferentiation *in vitro*. As suppressors of the *CDH1* promoter^22^, ZEB1 and ZEB2 are likely to play an active role in loss of the epithelial phenotype in expanding BCD cells^7^. ZEB proteins have been reported as downstream effectors of major EMT-inducing pathways, some of which already reported by our group to drive EMT during *ex vivo* expansion of BCD cells. Reduction in E-Cadherin levels controlled by the WNT-β-catenin, TGFβ-SMAD2/3, and NOTCH pathways is mediated by activation of the transcription repressors SNAIL1 and SNAIL2 (SLUG)^12^,^13^,^34^,^35^,^36^, which induce expression of the *ZEB* genes^9^. *ZEB1* was also found to be induced by TGFβ through SMAD3^37^. In addition, *ZEB1* transcription is activated by β-catenin/TCF4, which binds directly to the ZEB1 promoter^38^. We have reported that the WNT-β-catenin and NOTCH pathways^20^,^21^, as well as SLUG^18^, are activated during islet cell propagation in culture.

We show that inhibition of ZEB1 expression is sufficient for induction of β-cell gene expression, MET, and growth arrest. These effects, which are reproducible in cells derived from multiple human donors, represent redifferentiation of BCD cells, rather than *de-novo* differentiation of other cell types present in the islet cell culture. Inhibition of ZEB1 expression also potentiated RC-induced redifferentiation, as judged by an improved response to glucose stimulation and a 3-fold increase in the fraction of C-peptide-positive cells to 60% of BCD cells. This is the highest redifferentiation rate achieved so far, compared with other treatments^18^,^20^,^21^,^29^. In contrast to ZEB1, ZEB2 inhibition led to minor effects, suggesting a less prominent role of this factor in BCD cell phenotype.

Our findings suggest that the effects of ZEB1 in BCD cells are likely mediated, at least in part, by the ZEB1/miR-200c feedback loop. miR-200 family expression has been identified in human islets and found to be highly downregulated during BCD cell dedifferentiation^29^. In rat INS-1 cells and mouse islets, miR-200 has been found to inhibit EMT by direct targeting of *Zeb1* transcripts, thereby increasing E-cadherin expression^39^. miR-200c levels increased more than 3-fold following ZEB1 inhibition. An increase in miR-200c levels upon RC treatment may explain the synergistic effect of combined *ZEB1* shRNA and RC treatments on BCD cell redifferentiation. Two miR-200c targets, *SOX6* and *SOX2*, are suggested as possible mediators of the miR-200c effects. Direct inhibition of SOX6 expression by shRNA led to an increase in insulin transcript levels, which is comparable to that achieved with ZEB1 inhibition. Similarly, *SOX2* shRNA upregulated *CDKN1*B transcript levels to a similar extent as ZEB1 inhibition. Conceivably, additional elements could be involved in mediating the ZEB1 effects.

Our findings demonstrate the key role of ZEB1 in EMT in cultured BCD cells, and suggest that ZEB1 inhibition may contribute to protocols of BCD cell redifferentiation, aimed at generating functional human β-like cells for cell therapy of diabetes. In addition, ZEB1 inhibition may contribute to reversing β-cell dedifferentiation *in vivo*^40^, which has been implicated in type 2 diabetes^41^.

## Methods

### Ethics statement

This study was conducted according to the principles expressed in the Declaration of Helsinki. The Institutional Review Boards of the following medical centers, which provided human islets, each provided approval for the collection of samples and subsequent analysis: University of Geneva School of Medicine; San Raffaele Hospital, Milan; Faculty of Medicine, Lille 2 University; Massachusetts General Hospital; University of Alberta, Canada; Washington University; University of Pennsylvania; Scharp/Lacy Institute; University of Illinois; University of Miami; Southern California Islet Consortium. All donors provided written consent for the collection of all samples and subsequent analysis.

### Cell culture

Human islets were received 2–4 days following isolation from individual donors (Supplementary Table S1). Islets were dissociated into single cells and cultured in CMRL 1066 medium containing 5.6 mM D-glucose and supplemented with 10% FCS (HyClone, Logan, UT), 100 U/ml penicillin, 100 μg/ml streptomycin, 100 μg/ml gentamycin, and 5 μg/ml amphotericine B (all tissue culture reagents were from Biological Industries, Israel) (growth medium) as described^5^. Culture medium was changed every 3–4 days. Once confluency was reached (day 7–10 in each passage), cells were detached by trypsin (Bio-Lab Ltd., Jerusalem, Israel) and reseeded at a 1:2 ratio. For redifferentiation, cells were trypsinized and seeded in ultra-low attachment plates with Redifferentiation Cocktail (RC) for 4–8 days as previously described^18^. For days 1–6 of RC treatment, CMRL 1066 medium containing 5.6 mM glucose and supplemented with 100 U/ml penicillin, 100 μg/ml streptomycin, 100 μg/ml gentamycin sulphate, 1% BSA fraction V (Sigma-Aldrich, St. Louis, MO), 1X ITS (Gibco BRL, Life Technologies, Grand Island, NY), D-glucose (at a final concentration of 25 mM), 8 nM exendin-4 (Acris, Herford, Germany), 8 nM activin A (Cytolab/PreproTech Asia, Rehovot, Israel), 1X B27 supplement (Stem Cell Technologies, Vancouver, Canada) and 10 mM Nicotinamide (Sigma-Aldrich). For days 7–8 of RC treatment, the same medium was used but without the addition of D-glucose and activin A. The medium was replaced every two days.

### Virus production, cell infection and cell sorting

RIP-Cre/ER and pTrip–loxP-Stop-loxP-eGFP lentiviruses^7^ were used for lineage tracing. Virus production, cell infection, and tamoxifen treatment were previously described^7^. eGFP-labeled cells were sorted using a FACS Aria sorter as described^6^,^7^. shRNA cloned in pLKO.1-PURO lentiviral vectors (TRCN-017563 and -017566 for *ZEB1*, TRCN-013528 for *ZEB2*, TRCN-355694 and -010772 for *SOX2*, TRCN-017988 and -017991 for *SOX6*, and scrambled control) were obtained from Sigma-Aldrich. For ZEB inhibition, cells were infected at MOI of 3:1 in CMRL 1066 medium containing 8 μg/ml polybrene (Sigma-Aldrich) overnight. Four days following infection the cells were selected with 1 μg/ml puromycin for 3 days. For miR-200c retrovirus production, the pre-mmu-miR-200c miR-VEC-Blasticidin vector^42^ was co-transfected into human embryonic kidney 293T cells with the Ampo-helper packaging plasmid. The medium was replaced 6 h post-transfection, and the virus was harvested 24 h later and used fresh. 1 × 10^6^ cells were plated in 14-cm culture dishes in growth medium for 24 h. Cells were infected at MOI of 3:1 in medium containing 8 μg/ml polybrene for 6 h. The infection was repeated two more times in the following two days. Selection was initiated 2–3 days later with 4 μg/ml blasticidin for 7 days. Following selection (a total of 10 days from the first infection), the cells were harvested for analysis.

### qPCR analyses

Total RNA was extracted using ZR RNA MiniPrepTM kit (Zymo Research, Irvine, CA) or TRIzol (Sigma-Aldrich), including DNase I (Thermo Scientific, Waltham, MA) treatment for removing genomic DNA. cDNA was prepared using High Capacity cDNA RT Kit (Applied Biosystems, Foster City, CA). qPCR was carried out using ABsolute blue qPCR Mix (Thermo Scientific) or FastStart Universal Probe Library Master Mix (Roche Life Science, Basel, Switzerland) in a 7300 real-time PCR instrument (Applied Biosystems). The results were normalized to transcripts of TATA-box-binding protein (TBP) and human large ribosomal protein (RPLPO)^43^ or glyceraldehyde-3-phosphate dehydrogenase (GAPDH). Supplementary Table S2 lists primer sequences. All reactions were performed with annealing at 60 °C for 40 cycles. For undetectable transcripts, the cycle number was set to 40 for comparisons. cDNA for miRNA analyses was prepared and analyzed using Taqman® MicroRNA Assay kit (Applied Biosystems) according to the manufacturer, with primers listed in Supplementary Table S3.

### Immunofluorescence analyses

Cells were trypsinyzed, spotted on slides using a Shandon Cytospin4 centrifuge (Thermo Scientific), and fixed for 15 min at RT in 4% paraformaldehyde (PFA). Samples were blocked for 30 min in PBS containing 1% BSA, 5% fetal goat serum (FGS), and 0.2% saponin (blocking buffer). Samples were incubated overnight at 4 °C with primary antibodies diluted in blocking buffer as follows: rat anti-human C-peptide (1:1000; BCBC); mouse anti-human PDX1 (1:500; R&D, Minneapolis, MN); mouse anti-NKX2.2 (1:1000; Hybridoma Bank, Iowa City, IA); mouse anti-NKX6.1 (1:1000; Hybridoma Bank); rabbit anti-eGFP (1:1000; Life Technologies, Carlsbad, CA); and mouse anti-Ki67 (1:200; Zymed, San Francisco, CA). Slides were washed in PBS with 0.1% Tween 20 (Sigma) and incubated with Alexa fluorophore-conjugated secondary antibodies (1:1000; Life Technologies). Nuclei were stained with DAPI. Images were obtained using a Leica SP5 confocal microscope.

### Cell apoptosis analyses

Apoptotic cells were detected by TUNEL staining using the *in situ* Cell Death Detection Kit (Roche). DNase I-treated specimen served as a positive control, according to manufacturer’s protocol.

### Immunoblotting

Total protein was extracted from cells by incubation with a lysis buffer containing 0.5% NP-40 and protease inhibitor cocktail for 10 min. 20–25 μg protein were resolved by SDS-PAGE and electroblotted onto Immobilon-P 0.45-μm membrane (Millipore, Billerica, MA), followed by incubation overnight at 4 °C with rabbit anti-ZEB1 (1:1000; Cell Signaling, Danvers, MA); mouse anti-ZEB2 (1:500; Abnova, Taipei City, Taiwan); rabbit anti-SOX6 (1:500; Abcam); or rabbit anti-SOX2 (1:1000; Cell Signaling). Mouse anti-HSC70 (1:1000; Santa Cruz Biotechnology, Dallas, TX) was used to monitor loading^44^ The bound antibody was visualized with the appropriate horseradish peroxidase-conjugated or biotin-conjugated anti-IgG (Jackson Immunoresearch, West Grove, PA), horseradish peroxidase-conjugated ExtrAvidin (for biotin-conjugated anti-IgG; Sigma-Aldrich), and SuperSignal West Pico chemiluminescent substrate (Thermo Scientific). Quantification was done using TINA 2.0 software.

### Human C-peptide and cell transplantation analyses

Cells were pre-incubated for 1 hour in Krebs–Ringer buffer (KRB), followed by incubation for 2 hours in KRB containing 0.5 mM 1-isobutyl 3-methylxanthine (IBMX) and 16.7 mM glucose. C-peptide content was determined in acidic alcohol cell extract. Human C-peptide was quantified using an ultrasensitive ELISA kit (Mercodia, Uppsala, Sweden; sensitivity 1.5 pmol/L; crossreactivity with insulin and proinsulin 0.0006% and 1.8%, respectively) according to the manufacturer’s protocol. 1–2 × 10^6^ cells were injected with a 30-gauge needle under the kidney capsule of >12 week-old nonobese diabetic severe combined immunodeficient IL2Rγ^-/-^ (NOD-SCID-gamma, NSG) mice. Human C-peptide was quantified in serum samples obtained from the facial vein of fed mice. Serum obtained from untreated mice was used as control.

### cDNA microarray analysis

Hybridization to Affymetrix GeneChip Human Gene 1.1 ST Arrays, washing, and scanning were performed according to the manufacturer (Affymetrix, Santa Clara, CA). Data analysis was performed on CEL files using Partek^®^ Genomics Suite TM (Partek Inc., St. Louis, MO). Data were normalized with the multi-average method^45^. Batch effect removal was applied for the different samples, followed by one-way ANOVA. Clustering analysis was performed by Partek Genomics Suite software with Pearson’s dissimilarity correlation by average linkage methods. Functional annotation was performed using the DAVID software. The raw data was deposited in the GEO database (accession number GSE69658).

### Statistical analyses

Significance was determined using two-tailed t-test. To approach a normal distribution, a logarithmic transformation was performed. To account for multiple testing, the Bonferroni correction was applied.

## Additional Information

**How to cite this article**: Sintov, E. *et al.* Inhibition of ZEB1 expression induces redifferentiation of adult human β cells expanded *in vitro*. *Sci. Rep.* **5**, 13024; doi: 10.1038/srep13024 (2015).

## Supplementary Material

Supplementary figures and tables

## Figures and Tables

**Table 1:** Differentially expressed genes selected for qPCR validation of microarray results.

	Gene Symbol	Gene Assignment	p-Value	Fold-Change
Up-regulated	*TMEM2*	Transmembrane protein 2	4.0E-05	3.40
	*CLDN1*	Claudin 1	2.5E-03	2.75
	*F11R*	F11 receptor	2.6E-05	2.23
	*ITGA3*	Integrin, alpha 3 (CD49C)	3.1E-05	1.87
	*OCLN*	Occludin	4.6E-03	1.71
	*INADL*	InaD-like (Drosophila) (PATJ)	1.7E-03	1.65
Down-regulated	*MTOR*	mechanistic target of rapamycin (serine/threonine kinase)	3.6E-06	−1.77
	*VCL*	Vinculin	1.3E-06	−1.83
	*CETN2*	Centrin, EF-hand protein, 2	3.3E-08	−2.75
	*LMOD1*	Leiomodin 1 (smooth muscle)	5.7E-05	−3.43
