# Supplementary material for: Inhibition of ZEB1 expression induces redifferentiation of adult human β cells expanded in vitro
Source: Sci Rep. 2015 Aug 12;5:13024. doi: 10.1038/srep13024 (PMC4532995; doi:10.1038/srep13024)
Supplement: Supplementary figures and tables [file srep13024-s1.pdf]

Inhibition of ZEB1 expression induces redifferentiation of adult human β cells expanded in vitro

Elad Sintov, Gili Nathan, Sarah Knoller, Metsada Pasmanik-Chor, Holger A. Russ, and Shimon Efrat

**Supplementary Table S1. Islet donors used in this study**

| Donor number | Donor sex | Donor age (y) | Donor BMI | Islet purity (%) |
|---|---|---|---|---|
| 1 | f | 44 | 21.5 | 85 |
| 2 | m | 40 | 29 | 95 |
| 3 | f | 47 | 33.2 | 70 |
| 4 | f | 62 | 31.5 | 90 |
| 5 | m | 58 | 43.0 | 80 |
| 6 | f | 27 | 23.4 | 70 |
| 7 | f | 65 | 27.3 | 90 |
| 8 | m | 59 | 23 | 85 |
| 9 | m | 54 | 24.3 | 90 |
| 10 | f | 63 | 23.4 | 70 |
| 11 | m | 29 | 30.2 | 95 |
| 12 | f | 42 | 32.5 | 93 |
| 13 | m | 34 | 27.8 | 85 |
| 14 | m | 64 | 24.2 | 80 |
| 15 | f | 39 | 21.9 | 90 |
| 16 | m | 56 | 30.8 | 90 |

| | | | | |
|---|---|---|---|---|
| 17 | f | 46 | 33 | 80 |
| 18 | m | 54 | 33 | 85 |
| 19 | m | 47 | 33.2 | 90 |
| 20 | m | 62 | 26 | 90 |
| 21 | f | 60 | 26.4 | 90 |
| 22 | f | 48 | 22 | 95 |
| 23 | m | 65 | 23.5 | 85 |
| 24 | m | 46 | 24 | 74 |
| 25 | m | 54 | 26.7 | 75 |
| 26 | m | 48 | 18 | 90 |
| 27 | m | 45 | 26.7 | 85 |
| 28 | m | 59 | 24.5 | 85 |
| 29 | f | 20 | 24.6 | 85 |
| 30 | f | 51 | 21.2 | 85 |
| 31 | m | 53 | 24.5 | 95 |
| 32 | m | 55 | 33.6 | 92 |
| 33 | m | 43 | 34.7 | 80 |
| 34 | f | 47 | 20.6 | 90 |
| 35 | m | 31 | 29 | 85 |
| 36 | f | 55 | 24.2 | 80 |
| 37 | m | 14 | 27.1 | 80 |
| 38 | m | 27 | 20.2 | 85 |
| 39 | m | 32 | 25.7 | 70 |

| | | | | |
|---|---|---|---|---|
| 40 | m | 52 | 21.2 | 80 |
| 41 | f | 37 | 23.8 | 80 |
| 42 | f | 32 | 27.4 | 80 |
| 43 | f | 38 | 33.1 | 90 |
| 44 | f | 47 | 22.5 | 70 |
| 45 | f | 47 | 25 | 70 |
| 46 | f | 61 | 31.1 | 90 |
| 47 | f | 45 | 34.3 | 80 |
| 48 | f | 52 | 31.4 | 80 |
| 49 | m | 48 | 30.7 | 80 |
| 50 | m | 15 | 23 | 90 |
| Mean±SD | | 46±13 | 27±5 | 84±7 |

**Supplementary Table S2. List of primers used in qPCR analyses**

| Gene | Sense primer | Antisense primer |
|---|---|---|
| *ACTA2* | GCTTTCAGCTTCCCTGAACA | GGAGCTGCTTCACAGGATTC |
| *CDH1* | GCCGAGAGCTACACGTTCA | GACCGGTGCAATCTTCAAA |
| *CDH2* | CTCCATGTGCCGGATAGC | CGATTTCACCAGAAGCCTCTAC |
| *CDKN1A* | CCGAAGTCAGTTCCTTGTGG | CATGGGTTCTGACGGACAT |
| *CDKN1B* | TTTGACTTGCATGAAGAGAAGC | AGCTGTCTCTGAAAGGGACATT |
| *CETN2* | AGGATGAGTTCTTTAGCTTTGCTT | AATAGGCTGCCTTCAACACAA |
| *CLDN1* | CCTATGACCCCAGTCAATGC | ACAGCAAAGTAGGGCACCTC |
| *F11R* | GCAGCCGTCCTTGTAACC | GGCTGGCTGTAAATCACCTT |
| *GCG* | GTACAAGGCAGCTGGCAAC | TGGGAAGCTGAGAATGATCTG |
| *HLXB9* | TGCCTAAGATGCCCGACTT | AGCTGCTGGCTGGTGAAG |
| *IAPP* | TTACCAAATTGTAGAGGCTTTCG | CCCTGCCTCTATACACTCACTACC |
| *INADL* | ACCCCTCGGTCTTAGGAAAA | TTGCTGATCTCTGCCGTGT |
| *INS* | AGGCTTCTTCTACACACCCAAG | CACAATGCCACGCTTCTG |
| *ITGA3* | CAACCAGGATGGATTTCAGG | CAGTCCCAGCTTCTCTCCAT |
| *LMOD1* | GGAAGATGGGAGACAAAGTCC | ACTGAAGCAGTTTGGGCACT |
| *MAFA* | AGCGAGAAGTGCCAACTCC | TTGTACAGGTCCCGCTCTTT |
| *MTOR* | AGCACCATCAACCTCCAAAA | GGCGATGATGAGTCCTTCAG |
| *NEUROD1* | CTGCTCAGGACCTACTAACAACAA | GTCCAGCTTGGAGGACCTT |
| *NKX2-2* | CGAGGGCCTTCAGTACTCC | GGGGACTTGGAGCTTGAGT |
| *NKX6-1* | CGTTGGGGATGACAGAGAGT | CGAGTCCTGCTTCTTCTTGG |
| *OCLN* | AGGAACCGAGAGCCAGGT | TGAGCAATGCCCTTTAGCTT |
| *PPY* | TCTAGTGCCCATTTACTCTGGAC | GCAGGTGGACAGGAGCAG |
| *PDX1* | CACATCCCTGCCCTCCTAC | GAAGAGCCGGCTTCTCTAAAC |
| *SST* | ACCCCAGACTCCGTCAGTTT | ACAGCAGCTCTGCCAAGAAG |
| *SOX2* | GGGGGAATGGACCTTGTATAG | GCAAAGCTCCTACCGTACCA |
| *SOX6* | AAAGGCAATTTACCAGTGATTTCT | CAAATGGAGAGGTGGCTTG |
| *TMEM2* | TCACTCTTCTTCCCTGTTCTGA | ACCCATGTGCAGGAACTGA |
| *VCL* | GGAGGTGATTAACCAGCCAAT | AATGATGTCATTGCCCTTGC |
| *ZEB1* | GCCAACAGACCAGACAGTGTT | TCTTGCCCTTCCTTTCCTG |

| | | |
|---|---|---|
| *ZEB2* | AAGCCAGGGACAGATCAGC | CCACACTCTGTGCATTTGAACT |

**Supplementary Table S3. List of primers used in miRNA analyses**

| Gene | Sequence | Assay no. |
|---|---|---|
| hsa-miR-200a | UAACACUGUCUGGUAACGAUGU | 000502 |
| hsa-miR-200b | UAAUACUGCCUGGUAAUGAUGA | 002251 |
| hsa-miR-200c | UAAUACUGCCGGGUAAUGAUGGA | 002300 |
| hsa-miR-24 | UGGCUCAGUUCAGCAGGAACAG | 000402 |
| U6 snRNA | TGCTCGCTTCGGCAGCACATATACTAAAATTGGAA CGATACAGAGAAGATTAGCATGGCCCCTGCGCAAG GATGACACGCAAATTCGTGAAGCGTTCCATATTTT | 001973 |

**Supplementary Table S4. Differentially expressed genes with largest change in expression**

| | Gene Symbol | Gene Assignment | p-Value | Fold-Change |
|---|---|---|---|---|
| Up-regulated | *LINGO2* | Leucine rich repeat and Ig domain containing 2 | 2.14E-05 | 5.33 |
| | *SPP1* | Secreted phosphoprotein 1 | 0.001471 | 5.11 |
| | *TMEM2* | Transmembrane protein 2 | 4.01E-05 | 3.40 |
| | *ACTBL2* | Actin, beta-like 2 | 1.04E-05 | 3.34 |
| | *MIR503* | microRNA 503 | 3.97E-05 | 3.28 |
| | *HIST2H4B* | Histone cluster 2, H4b | 4.85E-06 | 3.18 |
| | *MT1F* | Metallothionein 1F | 0.000549 | 3.14 |
| | *SLITRK6* | SLIT and NTRK-like family, member 6 | 9.85E-05 | 3.08 |
| | *PRR15* | Proline rich 15 | 2.45E-05 | 3.06 |
| | *ABCC3* | ATP-binding cassette, sub-family C (CFTR/MRP), member 3 | 1.15E-09 | 2.89 |
| Down-regulated | *SCRG1* | Stimulator of chondrogenesis 1 | 3.19E-06 | -2.87 |
| | *KRTAP1-5* | Keratin associated protein 1- | 7.75E-05 | -2.88 |
| | *ZEB1* | Zinc finger E-box binding homeobox 1 | 4.43E-07 | -2.91 |
| | *SYT11* | Synaptotagmin XI | 1.01E-05 | -3.02 |
| | *TNC* | Tenascin C | 1.02E-05 | -3.02 |
| | *MYOCD* | Myocardin | 1.16E-05 | -3.06 |

| | | | | |
|---|---|---|---|---|
| | *LOXL4* | Lysyl oxidase-like | 0.000216 | -3.10 |
| | *WRB* | Tryptophan rich basic protein | 1.06E-06 | -3.16 |
| | *NTN4* | Netrin 4 | 8.53E-06 | -3.16 |
| | *LMOD1* | Leiomodin 1 (smooth muscle) | 5.7E-05 | -3.43 |

**Supplementary Table S5. DAVID functional annotation analysis**

| | GO term category number | GO term | # of genes | p-Value |
|---|---|---|---|---|
| **Up-regulated** | GO:0045449 | regulation of transcription | 216 | 1.01E-08 |
| | GO:0015837 | amine transport | 20 | 4.97E-05 |
| | GO:0006865 | amino acid transport | 17 | 5.68E-05 |
| | GO:0046942 | carboxylic acid transport | 21 | 3.42E-04 |
| | GO:0015697 | quaternary ammonium group transport | 6 | 3.74E-04 |
| | GO:0015849 | organic acid transport | 21 | 3.74E-04 |
| | GO:0031497 | chromatin assembly | 13 | 4.48E-03 |
| | GO:0015804 | neutral amino acid transport | 6 | 6.20E-03 |
| | GO:0015695 | organic cation transport | 6 | 9.31E-03 |
| | GO:0035195 | gene silencing by miRNA | 5 | 1.49E-02 |
| | GO:0045941 | positive regulation of transcription | 45 | 2.88E-02 |
| | GO:0034329 | cell junction assembly | 7 | 3.03E-02 |
| | GO:0035194 | posttranscriptional gene silencing by RNA | 5 | 3.11E-02 |
| | GO:0016441 | posttranscriptional gene silencing | 5 | 3.11E-02 |
| | GO:0010608 | posttranscriptional regulation of gene expression | 20 | 3.96E-02 |
| | GO:0005911 | cell-cell junction | 20 | 4.56E-03 |
| | GO:0043296 | apical junction complex | 13 | 5.01E-03 |
| | GO:0008270 | zinc ion binding | 195 | 5.26E-08 |
| | GO:0046914 | transition metal ion binding | 224 | 1.88E-07 |
| | GO:0043167 | ion binding | 316 | 3.00E-07 |
| | GO:0046872 | metal ion binding | 309 | 3.87E-07 |
| | GO:0043169 | cation binding | 310 | 6.95E-07 |

| | | | | |
|---|---|---|---|---|
| | GO:0015175 | neutral amino acid transmembrane transporter activity | 7 | 1.05E-03 |
| | GO:0015294 | solute:cation symporter activity | 15 | 1.32E-03 |
| | GO:0015171 | amino acid transmembrane transporter activity | 11 | 2.20E-03 |
| | GO:0051119 | sugar transmembrane transporter activity | 7 | 3.46E-03 |
| | GO:0005351 | sugar:hydrogen symporter activity | 5 | 1.24E-02 |
| | GO:0005402 | cation:sugar symporter activity | 5 | 1.24E-02 |
| | GO:0015179 | L-amino acid transmembrane transporter activity | 7 | 2.28E-02 |
| | GO:0035257 | nuclear hormone receptor binding | 10 | 3.71E-02 |
| **Down-regulated** | GO:0031012 | extracellular matrix | 42 | 3.53E-04 |
| | GO:0044430 | cytoskeletal part | 93 | 3.70E-04 |
| | GO:0044421 | extracellular region part | 93 | 4.87E-04 |
| | GO:0015630 | microtubule cytoskeleton | 59 | 5.59E-04 |
| | GO:0043292 | contractile fiber | 19 | 1.40E-03 |
| | GO:0005578 | proteinaceous extracellular matrix | 35 | 6.73E-03 |
| | GO:0015629 | actin cytoskeleton | 29 | 1.67E-02 |
| | GO:0005876 | spindle microtubule | 6 | 4.44E-02 |
| | GO:0005615 | extracellular space | 59 | 4.96E-02 |
| | GO:0051301 | cell division | 42 | 1.73E-06 |
| | GO:0000187 | activation of MAPK activity | 16 | 1.77E-04 |
| | GO:0022403 | cell cycle phase | 46 | 2.81E-04 |
| | GO:0000278 | mitotic cell cycle | 41 | 6.71E-04 |
| | GO:0007010 | cytoskeleton organization | 46 | 8.54E-04 |
| | GO:0022402 | cell cycle process | 55 | 1.68E-03 |
| | GO:0007155 | cell adhesion | 65 | 1.87E-03 |

| | | | | |
|---|---|---|---|---|
| | GO:0022610 | biological adhesion | 65 | 2.01E-03 |
| | GO:0051302 | regulation of cell division | 10 | 2.47E-03 |
| | GO:0051781 | positive regulation of cell division | 9 | 2.70E-03 |
| | GO:0034330 | cell junction organization | 11 | 2.86E-03 |
| | GO:0007049 | cell cycle | 68 | 5.97E-03 |
| | GO:0042127 | regulation of cell proliferation | 68 | 7.96E-03 |
| | GO:0051272 | positive regulation of cell motion | 14 | 8.91E-03 |
| | GO:0030335 | positive regulation of cell migration | 13 | 1.03E-02 |
| | GO:0051270 | regulation of cell motion | 22 | 1.10E-02 |
| | GO:0007254 | JNK cascade | 10 | 1.15E-02 |
| | GO:0008283 | cell proliferation | 41 | 1.20E-02 |
| | GO:0030334 | regulation of cell migration | 19 | 2.15E-02 |
| | GO:0045104 | intermediate filament cytoskeleton organization | 5 | 3.46E-02 |

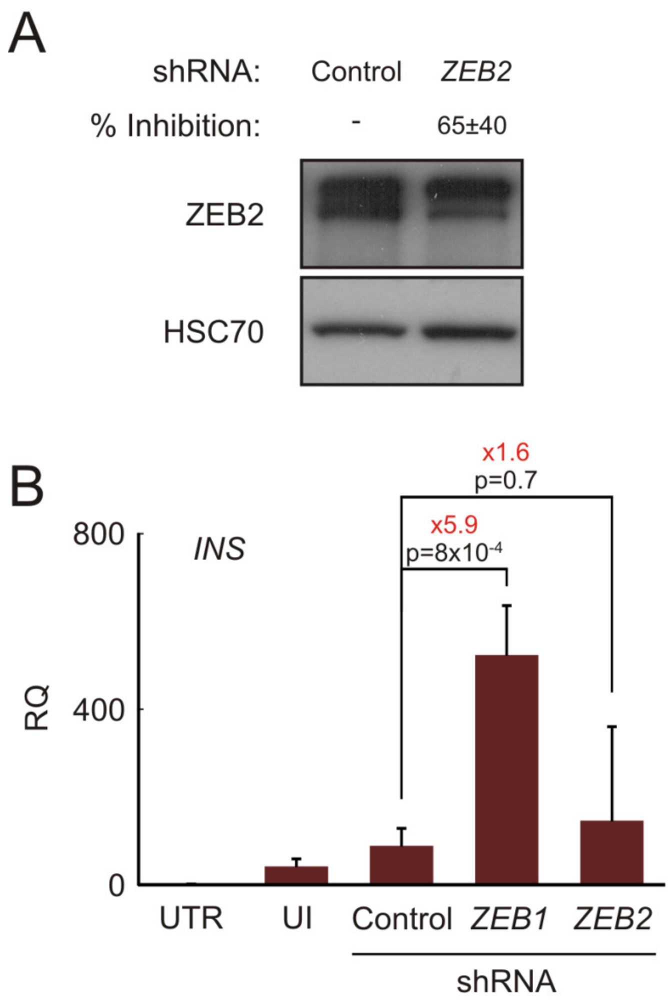


**Supplementary Fig. S1. Comparison of the effects of ZEB1 and ZEB2 inhibition on insulin expression in expanded islet cells. A**, ZEB2 inhibition by shRNA. Immunoblotting analysis of expanded islet cells infected at passage 6 with lentiviruses expressing *ZEB2* or control shRNA and analyzed 7 days later. **B**, qPCR analysis of *INS* transcripts in RNA extracted from expanded islet cells 7 days following infection at passages 5-8 with lentiviruses expressing *ZEB1* shRNA#1, *ZEB2* shRNA, or control shRNA, and a 4-day treatment with RC. Values are mean±SE (n=3-6 donors), relative to untreated (UTR) cells, and normalized to human *RPLPO* and *TBP*. UI, uninfected cells treated with RC.

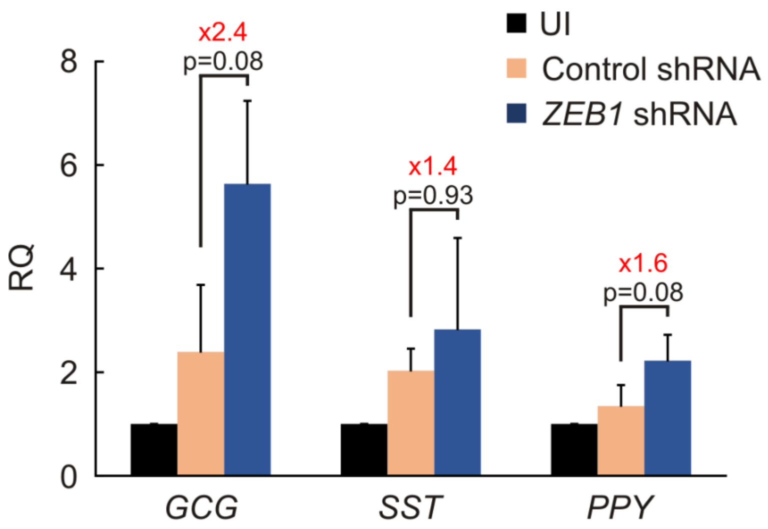


**Supplementary Fig. S2. Effect of ZEB1 inhibition on expression of transcripts encoding islet hormones in BCD cells.** Expanded islet cells transduced with the lineage tracing lentiviruses were sorted by FACS at passages 2-3. eGFP-labeled BCD cells were then grown until passages 6-7 and transduced with lentiviruses expressing *ZEB1* or control shRNAs. RNA was extracted 7 days later and analyzed by qPCR. Values are mean±SE (n=3-4 donors), relative to uninfected (UI) cells, and normalized to human *RPLPO* and *TBP*.

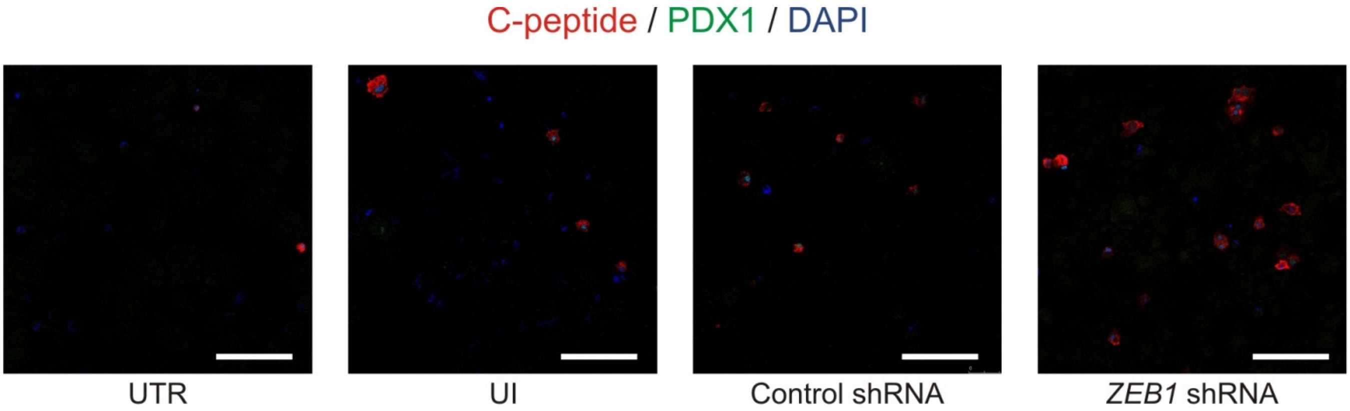


**Supplementary Fig. S3. Effect of ZEB1 inhibition and RC treatment on the number of cells co-stained with C-peptide and PDX1.** Immunofluorescense analysis of expanded islet cells, 7 days following infection at passages 5 with lentiviruses expressing *ZEB1* or control shRNAs, and a 4-day treatment with RC. Bar=100 µm.

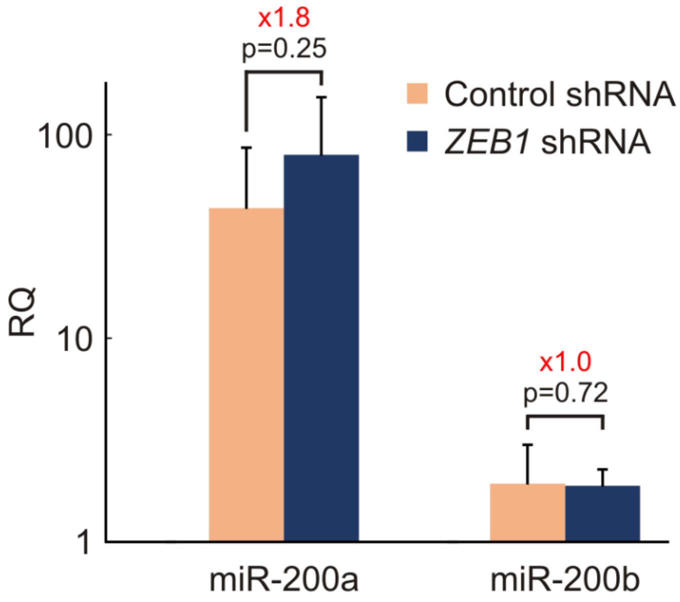


**Supplementary Fig. S4. Effect of ZEB1 inhibition on miR-200 expression in expanded BCD cells**. qPCR analysis of miR-200 expression in FACS-sorted BCD cells infected at passages 6-7 with lentiviruses expressing *ZEB1* or control shRNAs. Values are mean±SE (n=3 donors), relative to uninfected cells (RQ=1), and normalized to human U6 snRNA and miR-24.
